# Supplementary material for: Hot spot prediction in protein-protein interactions by an ensemble system
Source: BMC Syst Biol. 2018 Dec 31;12(Suppl 9):132. doi: 10.1186/s12918-018-0665-8 (PMC6311905; doi:10.1186/s12918-018-0665-8)
Supplement: Supplementary file 2 — All relevant MATLAB code. A simple Matlab implement of our predictor. (ZIP 19,742 kb) [file 12918_2018_665_MOESM2_ESM.zip › Additional file 2/Read ASA/ASA website/SKEMPI/1IARA.htm]

 NetSurfP - Protein Surface Accessibility and Secondary Structure Predictions

  

|  |  |  |
| --- | --- | --- |
|  |  | NetSurfP - Protein Surface Accessibility and Secondary Structure Predictions  Technical University of Denmark |

  


---

```
# For publication of results, please cite:
# A generic method for assignment of reliability scores applied to solvent accessibility predictions.
# Bent Petersen, Thomas Nordahl Petersen, Pernille Andersen, Morten Nielsen and Claus Lundegaard
# BMC Structural Biology 2009, 9:51 doi:10.1186/1472-6807-9-51
#
# Column 1: Class assignment - B for buried or E for Exposed - Threshold: 25% exposure, but not based on RSA
# Column 2: Amino acid
# Column 3: Sequence name
# Column 4: Amino acid number
# Column 5: Relative Surface Accessibility - RSA
# Column 6: Absolute Surface Accessibility
# Column 7: Z-fit score for RSA prediction
# Column 8: Probability for Alpha-Helix
# Column 9: Probability for Beta-strand
# Column 10: Probability for Coil
E H  1IARA                  1    0.758 137.862  -0.256   0.003   0.003   0.994
E K  1IARA                  2    0.614 126.403  -0.307   0.109   0.005   0.886
B C  1IARA                  3    0.216  30.256  -1.757   0.246   0.004   0.750
E D  1IARA                  4    0.669  96.388  -0.448   0.246   0.004   0.750
E I  1IARA                  5    0.499  92.389  -0.025   0.502   0.002   0.495
E T  1IARA                  6    0.328  45.494   1.006   0.600   0.003   0.397
B L  1IARA                  7    0.059  10.821   0.642   0.923   0.002   0.076
E Q  1IARA                  8    0.449  80.156   1.531   0.970   0.001   0.030
E E  1IARA                  9    0.465  81.166   1.092   0.970   0.001   0.030
B I  1IARA                 10    0.040   7.419   0.161   0.970   0.001   0.030
B I  1IARA                 11    0.084  15.540   0.533   0.970   0.001   0.030
E K  1IARA                 12    0.483  99.394   0.992   0.970   0.001   0.030
B T  1IARA                 13    0.174  24.092   0.340   0.923   0.002   0.076
B L  1IARA                 14    0.066  12.140   0.480   0.858   0.002   0.139
E N  1IARA                 15    0.382  55.910  -0.143   0.858   0.002   0.139
E S  1IARA                 16    0.441  51.709   0.920   0.782   0.003   0.216
B L  1IARA                 17    0.103  18.878  -0.130   0.802   0.014   0.185
B T  1IARA                 18    0.279  38.642   0.345   0.622   0.015   0.363
E E  1IARA                 19    0.690 120.473  -0.136   0.339   0.016   0.645
E Q  1IARA                 20    0.616 109.946   0.663   0.181   0.016   0.803
E K  1IARA                 21    0.663 136.400  -0.379   0.058   0.017   0.925
E T  1IARA                 22    0.322  44.689  -0.095   0.018   0.047   0.935
E L  1IARA                 23    0.403  73.753   0.295   0.052   0.084   0.864
B C  1IARA                 24    0.116  16.329  -1.647   0.064   0.216   0.721
E T  1IARA                 25    0.284  39.335   0.067   0.066   0.296   0.638
E E  1IARA                 26    0.483  84.415  -0.778   0.148   0.418   0.435
B L  1IARA                 27    0.191  34.935   0.321   0.135   0.317   0.548
E T  1IARA                 28    0.301  41.749  -0.488   0.231   0.330   0.439
B V  1IARA                 29    0.155  23.747  -0.116   0.307   0.165   0.527
E T  1IARA                 30    0.366  50.764  -0.312   0.386   0.097   0.517
E D  1IARA                 31    0.444  63.966  -1.127   0.386   0.097   0.517
B I  1IARA                 32    0.180  33.282  -0.404   0.428   0.171   0.402
B F  1IARA                 33    0.213  42.809  -0.900   0.428   0.171   0.402
E A  1IARA                 34    0.401  44.179  -0.660   0.307   0.165   0.527
B A  1IARA                 35    0.243  26.757  -0.546   0.199   0.152   0.649
E S  1IARA                 36    0.431  50.490  -0.311   0.052   0.084   0.864
E K  1IARA                 37    0.577 118.730  -0.203   0.053   0.043   0.903
E N  1IARA                 38    0.529  77.446  -1.193   0.053   0.043   0.903
B T  1IARA                 39    0.291  40.376  -0.663   0.113   0.043   0.844
E T  1IARA                 40    0.415  57.533  -0.390   0.268   0.043   0.689
E E  1IARA                 41    0.444  77.584  -0.512   0.455   0.046   0.498
E K  1IARA                 42    0.439  90.220   0.066   0.660   0.049   0.291
E E  1IARA                 43    0.399  69.653  -0.229   0.751   0.050   0.199
B T  1IARA                 44    0.166  23.094   0.067   0.831   0.044   0.125
B F  1IARA                 45    0.093  18.685   0.575   0.831   0.044   0.125
B C  1IARA                 46    0.066   9.266   0.254   0.831   0.044   0.125
E R  1IARA                 47    0.327  74.906   0.899   0.879   0.010   0.111
B A  1IARA                 48    0.074   8.166  -0.816   0.938   0.007   0.055
B A  1IARA                 49    0.096  10.546   0.127   0.975   0.003   0.022
E T  1IARA                 50    0.382  52.997   0.569   0.975   0.003   0.022
B V  1IARA                 51    0.136  20.980  -0.159   0.975   0.003   0.022
B L  1IARA                 52    0.036   6.518   0.203   0.938   0.007   0.055
B R  1IARA                 53    0.241  55.166   0.563   0.938   0.007   0.055
E Q  1IARA                 54    0.398  71.011   1.252   0.938   0.007   0.055
B F  1IARA                 55    0.082  16.518   0.334   0.879   0.010   0.111
B Y  1IARA                 56    0.104  22.203  -0.173   0.660   0.049   0.291
E S  1IARA                 57    0.321  37.609   0.200   0.354   0.048   0.598
E H  1IARA                 58    0.538  97.826   0.425   0.113   0.043   0.844
E H  1IARA                 59    0.328  59.718   0.290   0.053   0.043   0.903
E E  1IARA                 60    0.720 125.871  -0.399   0.052   0.084   0.864
B K  1IARA                 61    0.251  51.528  -0.306   0.056   0.142   0.802
E D  1IARA                 62    0.412  59.355  -1.090   0.125   0.227   0.648
E T  1IARA                 63    0.345  47.838  -0.694   0.199   0.152   0.649
E R  1IARA                 64    0.482 110.447   0.064   0.278   0.093   0.628
E C  1IARA                 65    0.302  42.443  -1.165   0.278   0.093   0.628
E L  1IARA                 66    0.297  54.307  -1.097   0.278   0.093   0.628
E G  1IARA                 67    0.612  48.133  -0.908   0.184   0.043   0.773
E A  1IARA                 68    0.582  64.081  -0.479   0.184   0.043   0.773
E T  1IARA                 69    0.527  73.164  -0.972   0.115   0.016   0.868
E A  1IARA                 70    0.445  48.995  -1.034   0.181   0.016   0.803
E Q  1IARA                 71    0.480  85.639  -0.661   0.268   0.043   0.689
E Q  1IARA                 72    0.292  52.098  -0.723   0.268   0.043   0.689
E F  1IARA                 73    0.296  59.508  -1.394   0.354   0.048   0.598
E H  1IARA                 74    0.482  87.658  -0.809   0.430   0.016   0.555
E R  1IARA                 75    0.524 120.042  -1.092   0.522   0.016   0.462
E H  1IARA                 76    0.412  74.979  -1.008   0.694   0.003   0.303
E K  1IARA                 77    0.499 102.644  -1.177   0.782   0.003   0.216
E Q  1IARA                 78    0.400  71.422  -0.110   0.782   0.003   0.216
B L  1IARA                 79    0.078  14.355  -0.639   0.923   0.002   0.076
B I  1IARA                 80    0.156  28.897   0.207   0.923   0.002   0.076
E R  1IARA                 81    0.450 103.027   0.744   0.970   0.001   0.030
B F  1IARA                 82    0.151  30.225   0.114   0.923   0.002   0.076
B L  1IARA                 83    0.041   7.525  -0.066   0.923   0.002   0.076
E K  1IARA                 84    0.326  67.058   0.804   0.923   0.002   0.076
E R  1IARA                 85    0.385  88.257   0.108   0.858   0.002   0.139
B L  1IARA                 86    0.059  10.858   0.522   0.782   0.003   0.216
B D  1IARA                 87    0.131  18.892  -0.781   0.717   0.014   0.269
E R  1IARA                 88    0.464 106.187  -0.229   0.622   0.015   0.363
E N  1IARA                 89    0.366  53.568  -1.519   0.522   0.016   0.462
B L  1IARA                 90    0.084  15.454  -0.272   0.430   0.016   0.555
E W  1IARA                 91    0.384  92.424  -0.635   0.430   0.016   0.555
E G  1IARA                 92    0.459  36.131  -1.177   0.430   0.016   0.555
B L  1IARA                 93    0.244  44.640  -1.303   0.339   0.016   0.645
E A  1IARA                 94    0.344  37.898  -0.735   0.181   0.016   0.803
E G  1IARA                 95    0.608  47.842   0.027   0.058   0.017   0.925
E L  1IARA                 96    0.405  74.082  -0.440   0.018   0.047   0.935
E N  1IARA                 97    0.674  98.732   0.083   0.018   0.088   0.893
E S  1IARA                 98    0.577  67.578  -0.318   0.018   0.088   0.893
B C  1IARA                 99    0.069   9.702  -0.628   0.004   0.138   0.858
E P  1IARA                100    0.531  75.377   0.530   0.019   0.141   0.840
E V  1IARA                101    0.298  45.818  -1.100   0.019   0.141   0.840
E K  1IARA                102    0.586 120.602  -0.626   0.052   0.084   0.864
E E  1IARA                103    0.497  86.756  -0.230   0.184   0.043   0.773
E A  1IARA                104    0.434  47.783  -1.067   0.268   0.043   0.689
E N  1IARA                105    0.453  66.319   0.323   0.354   0.048   0.598
E Q  1IARA                106    0.415  74.101   0.714   0.455   0.046   0.498
E S  1IARA                107    0.357  41.899   0.431   0.455   0.046   0.498
E T  1IARA                108    0.266  36.936   0.880   0.660   0.049   0.291
B L  1IARA                109    0.073  13.311   0.162   0.923   0.002   0.076
E E  1IARA                110    0.556  97.133   1.454   0.970   0.001   0.030
E N  1IARA                111    0.500  73.244   0.796   0.970   0.001   0.030
B F  1IARA                112    0.063  12.664   0.468   0.970   0.001   0.030
B L  1IARA                113    0.115  21.130  -0.228   0.970   0.001   0.030
E E  1IARA                114    0.589 102.933   0.806   0.970   0.001   0.030
E R  1IARA                115    0.350  80.150   0.902   0.970   0.001   0.030
B L  1IARA                116    0.053   9.741   0.474   0.988   0.000   0.012
E K  1IARA                117    0.382  78.557   1.287   0.970   0.001   0.030
E T  1IARA                118    0.460  63.747   0.860   0.970   0.001   0.030
B I  1IARA                119    0.110  20.405   0.025   0.970   0.001   0.030
B M  1IARA                120    0.127  25.413   0.364   0.923   0.002   0.076
E R  1IARA                121    0.520 119.011   0.800   0.923   0.002   0.076
E E  1IARA                122    0.521  90.984  -0.047   0.858   0.002   0.139
E K  1IARA                123    0.423  87.032   0.175   0.782   0.003   0.216
B Y  1IARA                124    0.264  56.502  -0.494   0.694   0.003   0.303
E S  1IARA                125    0.467  54.686  -0.429   0.600   0.003   0.397
E K  1IARA                126    0.631 129.735   0.881   0.430   0.016   0.555
E C  1IARA                127    0.286  40.140  -2.162   0.257   0.016   0.727
E S  1IARA                128    0.651  76.309  -1.550   0.109   0.005   0.886
E S  1IARA                129    0.895 104.859  -0.009   0.003   0.003   0.994

Explain the output.  Go back.
```

---
